# Diagnostic value of SAT-TB in smear-negative pulmonary tuberculosis: A diagnostic accuracy study

**DOI:** 10.1097/MD.0000000000040907

**Published:** 2024-12-13

**Authors:** Xiaoxiao Zhao, Kunping Cui, Lang Bai, Shanling Xu, Wei Liu, Jin Shang, Rili Mise, Wen Quan Li, Lin Wang, Wen Qiu Deng, Lingcheng Cheng, Chuan Zhao

**Affiliations:** a Center of Infectious Diseases, West China Hospital of Sichuan University, Chengdu, Sichuan, China; b Department of Medical Oncology, Sichuan Cancer Hospital & Institute, Sichuan Cancer Center, School of Medicine, University of Electronic Science and Technology of China, Chengdu, Sichuan, China.; c Infection Department, The Second People’s Hospital of Yibin, Yibin, Sichuan, China; d Infection Department, The People’s Hospital of Zhongjiang, Leshan, Sichuan, China; e Infection Department, The People’s Hospital of Mianzhu, Deyang, Sichuan, China; f Infection Department, Armed Police Forces Hospital of Sichuan, Leshan, Sichuan, China; g Infection Department, Suining Central Hospital, Suining, Sichuan, China

**Keywords:** diagnostic, SAT-TB, smear-negative pulmonary tuberculosis

## Abstract

This study aimed to evaluate the diagnostic value of rapid simultaneous RNA amplification and testing for tuberculosis (SAT-TB) in smear-negative pulmonary tuberculosis (PTB). We performed a multicenter prospective analysis of 206 patients with smear-negative suspected PTB between December 2018 and March 2022. We collected sputum or bronchoalveolar lavage fluid (BALF) for simultaneous SAT-TB and Xpert *Mycobacterium tuberculosis*/rifampin (MTB/RIF) assays. The efficiency of SAT-TB detection was also evaluated. The final analysis included 161 patients with smear-negative suspected PTB, of whom 114 provided sputum specimens and 47 provided BALF specimens. In sputum samples, the area under the curve, sensitivity, and specificity of SAT-TB for diagnosing PTB were 0.75, 50.7%, and 100.0%, respectively, and those of the Xpert MTB/RIF assay were 0.81, 62.3%, and 100.0%, respectively. The kappa coefficient k of the consistency between SAT-TB and Xpert MTB/RIF in sputum specimens was 0.686. In BALF specimens, the area under the curve, sensitivity, and specificity of SAT-TB for diagnosing PTB were 0.79, 57.1%, and 100.0%, respectively, and those of Xpert MTB/RIF were 0.86, 76.2%, and 96.2%, respectively. The kappa coefficient k of the consistency between SAT-TB and Xpert MTB/RIF in BALF specimens was 0.656. The SAT-TB and Xpert MTB/RIF assays were highly consistent in diagnosing smear-negative PTB. It is a valuable method for early detection, prevention, and managing smear-negative PTB suspects. Meanwhile, the detection efficiency and cost-effectiveness of SAT-TB are more suitable for the rapid diagnosis of smear-negative PTB in low- and middle-income countries.

## 1. Introduction

Pulmonary tuberculosis (PTB) is a chronic respiratory infectious disease caused by infection of the lungs by *Mycobacterium tuberculosis*. The World Health Organization predicted approximately 10.6 million new cases of tuberculosis and 1.6 million deaths globally in 2022, with PTB accounting for approximately 85% of the cases, thus posing a serious threat to global public health security. Although antituberculosis treatment is effective in more than 85% of cases, only 57% of global tuberculosis cases are diagnosed through bacteriology; the proportion is even lower in low- and middle-income countries, emphasizing the urgent need for early and rapid diagnosis of smear-negative PTB.^[1]^

In the face of a severe PTB epidemic, screening for the same is routinely conducted in many countries. In this context, the sputum smear test for anti-acid bacilli has become a commonly used method for primary screening of PTB because of its accessibility and low cost; however, its shortcomings include low sensitivity and specificity, inability to differentiate between *M tuberculosi*s and nontuberculous mycobacteria, a high rate of leakage and misdiagnosis, and a very limited diagnostic value for smear-negative PTB.^[2]^ Although patients with smear-negative PTB are usually considered noninfectious, about 17% of these patients with smear-negative culture-positive may still transmit the infection.^[3,4]^ Therefore, delayed diagnosis in these patients poses a risk for PTB lesion progression and public transmission. Although sputum culture examination can identify strains and improve the detection rate of smear-negative PTB, its shortcoming lies in the long culture period, which no longer meets the urgent clinical need for early and rapid diagnosis and treatment of the disease.^[5]^

In recent years, with the development of precision medicine, the Xpert *M tuberculosis*/rifampin (MTB/RIF) assay has become a new method that targets DNA detection and uses real-time fluorescence polymerase chain reaction to detect the 81 bp rifampicin resistance determination region of *M tuberculosi*s *rpoB* gene. The results can be obtained within a few hours, which can significantly shorten the detection time of smear-negative PTB, improve the sensitivity and specificity compared with culture detection technology, and is recommended by the World Health Organization. However, its shortcomings include high technical requirements, expensive equipment and equipment maintenance costs, high cost, and the inability to differentiate between live and dead bacilli; moreover, it has not been widely developed in many PTB high-burden countries and economically backward areas, thus further limiting the detection rate of smear-negative PTB.^[6–8]^ The simultaneous RNA amplification and testing for tuberculosis (SAT-TB) targets the pathogen 16S RNA, using RNA at 42 °C as the starting template and thermostatic amplification technology for RNA quantification, which can yield results within 2 hours. Compared with Xpert MTB/RIF, the SAT-TB method has the advantages of simple operation, low cost, less contamination, and the ability to identify live bacteria.^[9,10]^ Meanwhile, relevant literature has reported that SAT-TB is not inferior to Xpert MTB/RIF in terms of PTB diagnostic efficacy.^[11]^ Therefore, SAT-TB may be an alternative for the rapid diagnosis of PTB in countries with a high PTB burden for patients with smear-negative suspected PTB who are unable to undergo Xpert MTB/RIF testing.

In recent years, research on the rapid diagnosis of PTB has mainly focused on smear-positive, less or no sputum PTB; however, few studies have evaluated the diagnostic efficacy of SAT-TB and Xpert MTB/RIF in the same respiratory tract specimens of patients with smear-negative suspected PTB. This study aimed to evaluate the diagnostic value of SAT-TB in smear-negative, suspected PTB cases.

## 2. Materials and methods

### 2.1. Study design

This prospective multicenter study was conducted at the West China Hospital of Sichuan University, Second People’s Hospital of Yibin, People’s Hospital of Zhongjiang, People’s Hospital of Mianzhu, Armed Police Forces Hospital of Sichuan, and Suining Central Hospital. Patients with smear-negative suspected PTB who were hospitalized and untreated between December 2018 and March 2022 were included in the study. Patients who met the following criteria were considered to be suspected PTB: (1) cough, expectoration > 2 weeks, or suggestive PTB symptoms, such as hemoptysis in sputum; (2) chest X-rays/CT radiographic findings compatible with active TB, including plaques, nodules, streaks, or cavities; and (3) negative sputum anti-acid bacillus smears on 2 occasions, with a positive result from the tuberculin-purified protein derivative test and/or gamma-interferon release test.

The inclusion criteria were as follows: (1) suspected PTB; (2) age ≥ 18 years; (3) at least 2 consecutive negative sputum smear results; and (4) patients who were unable to submit competent sputum specimens were willing to undergo bronchoscopic lavage of lung lesions to obtain bronchoalveolar lavage fluid (BALF) specimens. Written informed consent was obtained from all patients or their guardians in accordance with the Declaration of Helsinki and the study was approved by the Biomedical Ethics Subcommittee of the West China Hospital of Sichuan University (approval number: 2018-340). The final clinical diagnosis was based on the “Health Industry Standard of the People’s Republic of China Tuberculosis Diagnosis” (WS 288-2017) as the reference standard. The “confirmed” PTB diagnosis was based on the detection of *M tuberculosis* on examination of respiratory specimens or a positive nucleic acid amplification test result, along with therapy response which the disease symptoms improved and the pulmonary lesions decreased after antituberculosis treatment. A diagnosis of “probable” PTB was made when the disease symptoms improved and the pulmonary lesions decreased after 2 weeks of diagnostic antituberculosis treatment despite negative culture, SAT-TB, and Xpert MTB/RIF test results, which after excluding other diseases with similar clinical manifestations, such as pneumonia, chronic obstructive pulmonary disease, bronchiectasis, lung cancer, and so on. In this study, the diagnoses of confirmed and probable PTB were regarded as the final clinical diagnoses. A diagnosis of non-PTB was made in cases where antituberculosis treatment was not initiated and other pulmonary diseases were diagnosed. The final “undetermined” diagnosis of PTB was established when (1) the patient recovered without receiving antituberculosis treatment despite a positive nucleic acid amplification test result and (2) nucleic acid amplification test results were negative and antituberculosis treatment was ineffective.

The exclusion criteria were as follows: (1) human immunodeficiency virus (HIV)-positive, (2) unclear final diagnosis, (3) failure to submit qualified sputum specimens, and unwillingness to undergo bronchoscopy, resulting in incomplete data.

### 2.2. Specimen collection

A total of 170 samples were obtained from patients with smear-negative suspected PTB, of which 121 were able to submit qualified sputum specimens did so after rinsing their mouths with clear water and coughing up approximately 9 to 10 mL deep sputum in the morning into a sterile specimen preservation tube, which was divided into 3 equal points on average. At the same time, 49 patients were unable to submit qualified sputum specimens, 40 to 60 mL volume of aseptic normal saline (0.9% NS) was injected into the airway of affected lung segment under a fiberoptic bronchoscope, and the collected 30 mL BALF specimens were divided into 3 equal parts.

To minimize the risk of RNA degradation, the following measures were taken: first, samples were stored in a refrigerated environment at 2 to 8 °C after collection. This temperature range helps to slow down the activity of RNA degradation enzymes. Second, aseptic handling techniques were used to prevent the degrading effect of RNase on RNA during sample processing and testing. In addition, all samples were tested within 24 hours of collection. The laboratory staff were blinded to the final diagnostic category.

### 2.3. Culture, SAT-TB, and Xpert MTB/RIF assays

Cultures were tested using the BACTEC MGIT 960 system (BD Diagnostic Systems, Sparks, MD) as follows: first, 2 mL of sputum or 10 mL of BALF was added to a 50 mL centrifuging tube; second, the same amount of 2% N-acetyl-L-cysteine–NaOH pretreatment solution was added to the tube, centrifuged for 20 seconds, and allowed to stand at room temperature for 15 to 20 minutes; third, add phosphate-buffered saline (PBS) to approximately 50 mL in the tube and centrifuged for 15 minutes; the fourth step involved discarding the supernatant after centrifugation and evenly mixing in 1 to 3 mL PBS to neutralize the pH to 6.8; and finally, 0.5 mL was removed and inoculated in a MGIT culture tube and the culture time was set to 42 days. If the instrument showed a positive result, it was a positive result, whereas if the instrument did not show a positive until the 42nd day, it was judged as negative.

The principle and process of SAT-TB assay was performed as previously published.^[9,12]^ The SAT-TB assays were performed according to the manufacturer’s instructions (Shanghai Rendu Biotechnology Co., China). The sample was prepared as follows: first, approximately 1.5 mL of sputum or BALF specimen was added to a 1.5 mL centrifuge tube, centrifuged at 14,170 r/min for 5 minutes, the supernatant discarded, and then 50 µL of *M tuberculosis*-RNA diluent was added, shaken well and finally resuspended in the same solution. Second, to extract the RNA, the test specimen and 50 μL of negative control were placed in the FZP-1 nucleic acid purifier (Shanghai Rendu Biotechnology Co., China), subjected to ultrasonic treatment for 15 minutes, centrifuged at 14170 r/min for 5 minutes, and the supernatant removed, which comprised the extracted RNA. Then, for amplification, 2 μL of the extracted RNA was added to a clean micro-reaction tube containing 30 μL amplification detection solution, which was placed in a K30 dry thermostat (Hangzhou Allsheng Instrument Co., China) at 60 °C for 10 minutes, followed by 42 °C for 5 minutes, and 10 μL SAT enzyme was then added while the sample was at 42 °C. The TL988 real-time fluorescent quantitative polymerase chain reaction instrument (Xi‘an Tianlong Technology Co., China) was used for amplification (amplification cycle: 42 °C for 1 minute, 40 cycles, 1 fluorescence per minute, FAM channel selected). The resulting cycle threshold (Ct) values of 40 and ≥ 40 were evaluated as MTB-RNA positive and MTB-RNA negative, respectively.

The Xpert MTB/RIF assay was performed in strict accordance with the manufacturer’s instructions (Cepheid, California). First, 1 mL specimen was placed in a sterile tube with a screw cap, treated with 2% N-acetyl-L-cysteine–NaOH and PBS, and centrifuged for later use; 2 mL of the treatment solution was added to the tube and the cap was tightened, vortexed for 10 seconds, and allowed to stand for 15 minutes at room temperature. Then, 2 mL of the treated specimen was placed in the GeneXpert MTB/RIF reaction kit into the detection module of the fully automated instrument.

### 2.4. Patient follow-up

All patients included in this study were followed up for at least 6 months, with equal assessment of the effectiveness of the antituberculosis treatment.

### 2.5. Statistical analysis

Continuous variables that conform to a normal distribution was expressed as mean ± standard deviation, and the Student *t* test was used for comparison between non-PTB and PTB groups. The count variables was expressed as n (%), and the χ^2^ test was used for comparison between non-PTB and PTB groups. The *P* value less than .05 was considered statistically significant.

To investigate the diagnostic efficacy of SAT-TB for smear-negative PTB, we used the final clinical diagnosis as the reference, the area under the curve (AUC), sensitivity, specificity, positive predictive value, and negative predictive value were calculated for the culture, SAT-TB, Xpert MTB/RIF, and the combined SAT-TB and Xpert MTB/RIF assay in different samples. The *Z* test was used to compare the AUC between SAT-TB and Xpert MTB/RIF. Meanwhile, Kappa analysis was used for consistency evaluation between SAT-TB and Xpert MTB/RIF.

SPSS 23.0 (IBM Corp, Armonk, NY) and MedCalc Statistical Software v15.2.2 (MedCalc Software bvba, Ostend, Belgium; http://www.medcalc.org) were used as the main software for data analysis.

## 3. Results

### 3.1. Study participants

**Figure 1.**
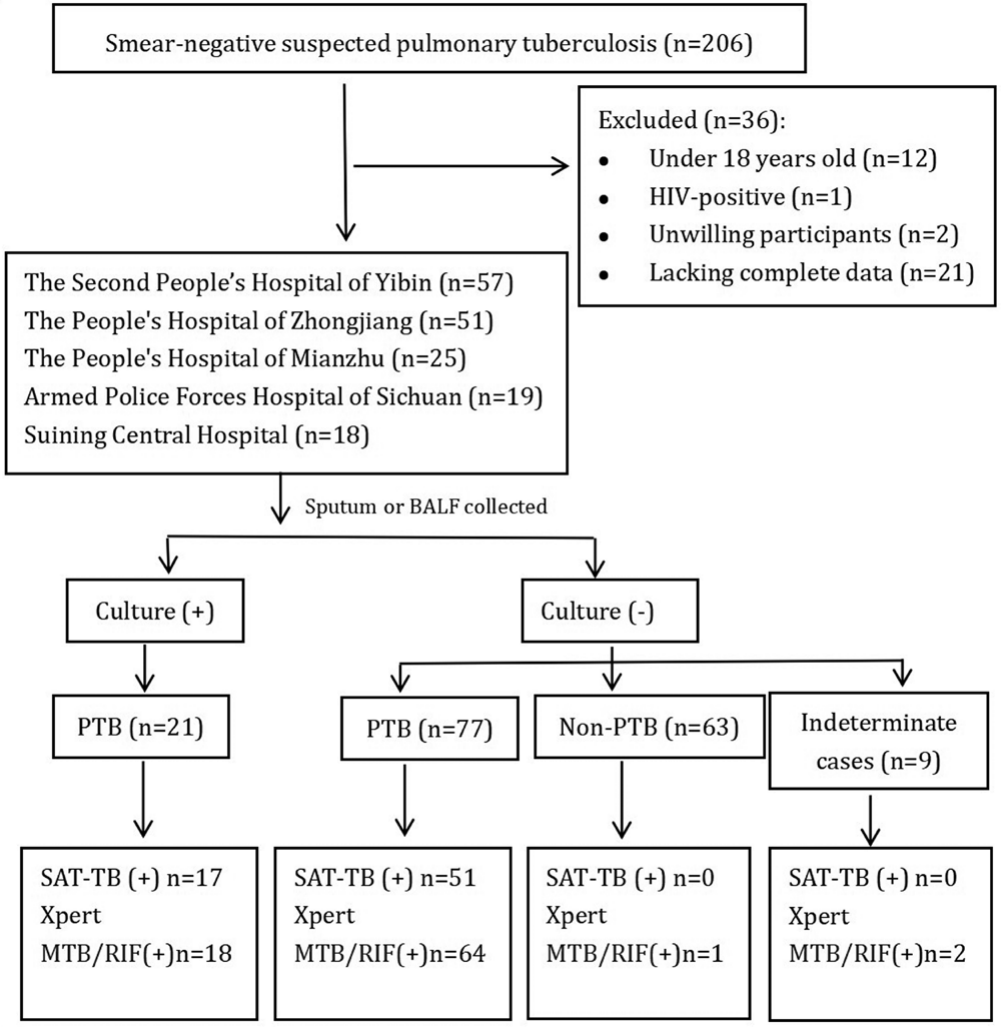
Categorization of patients included in the study. BALF = bronchoalveolar lavage fluid; culture = culture for *M tuberculosis*; indeterminate = lost to follow-up and unclear diagnosis; MTB/RIF = *Mycobacterium tuberculosis*/rifampicin; non-PTB = not pulmonary tuberculosis; PTB = pulmonary tuberculosis; SAT-TB = simultaneous RNA amplification and testing for tuberculosis.

In total, 206 potentially eligible patients with smear-negative suspected PTB were screened. After excluding patients younger than 18 years (n = 12), HIV-positive (n = 1), unwilling to participate (n = 2), with incomplete data (n = 21), and with undetermined diagnosis (n = 9), a total of 161 patients were included in the study. After comprehensive evaluations and follow-ups, 98 (60.87%) patients were diagnosed with PTB and 63 (39.13%) were non-PTB, one of whom was Xpert MTB/RIF-positive (previous history of PTB, no current evidence of tuberculosis activity) (Fig. 1).

### 3.2. Clinical characteristics of the study participants

In this study, men accounted for 71.4% (n = 115), the mean age was 49.0 ± 16.7 years, and most of the patients were aged 50 years and above (84/161, 52.2%). Clinical symptoms were mainly respiratory symptoms, such as cough, fever, and shortness of breath, which lacked specificity; however, night sweats, shortness of breath, and hemoptysis symptoms accounted for a significantly higher proportion of symptoms among patients with PTB than in those without PTB; thus, these patients need to be clinically monitored and further screened for PTB (Table 1).

**Table 1:** Demographic and clinical characteristics of the study participants.

	Total (n = 161)	Non-PTB (n = 63)	PTB (n = 98)	Statistical value	*P* value
Female (n, %)	115 (71.4)	37 (58.7)	78 (79.6)	8.178	.004
Age (years)	49.0 ± 16.7	51.3 ± 15.2	47.5 ± 17.6	1.418	.158
Specimen				7.303	.007
Sputum	114 (70.8)	37 (58.7)	77 (78.6)		
Bronchoalveolar lavage fluid	47 (29.2)	26 (41.3)	21 (21.4)		
Symptoms (n,%)					
Fever	52 (32.3)	15 (23.8)	37 (37.8)	3.411	.065
Cough	144 (89.4)	56 (88.9)	88 (89.8)	0.033	.855
Night sweats	21 (13.0)	0 (0)	21 (21.4)	15.525	<.001
Shortness of breath	48 (29.8)	9 (14.3)	39 (39.8)	11.926	.001
Hemoptysis	34 (21.1)	5 (7.9)	29 (29.6)	10.795	.001
Chest distress	36 (22.4)	17 (27.0)	19 (19.4)	1.275	.259
Weight loss	9 (5.6)	4 (6.3)	5 (5.1)	0.113	.737
Culture	21 (13.0)	0 (0)	21 (21.4)	15.525	<.001
SAT-TB	51 (31.7)	0 (0)	51 (52.0)	47.986	<.001
Xpert MTB/RIF	65 (40.4)	1 (1.6)	64 (65.3)	64.676	<.001

MTB/RIF = *Mycobacterium tuberculosis*/rifampin, SAT-TB = simultaneous RNA amplification and testing method for tuberculosis.

### 3.3. PTB detection by culture, SAT-TB, and Xpert MTB/RIF assays

Among the 98 patients with PTB, 21 were culture-positive, 17 were SAT-TB-positive, and 18 were Xpert MTB/RIF-positive. Of the 77 culture-negative non-PTB patients, 34 were SAT-TB-positive and 46 were Xpert MTB/RIF-positive. Culture, SAT-TB, and Xpert MTB/RIF test results were negative in 27 (27.55%) patients with PTB (Fig. 1).

**Figure 2.**
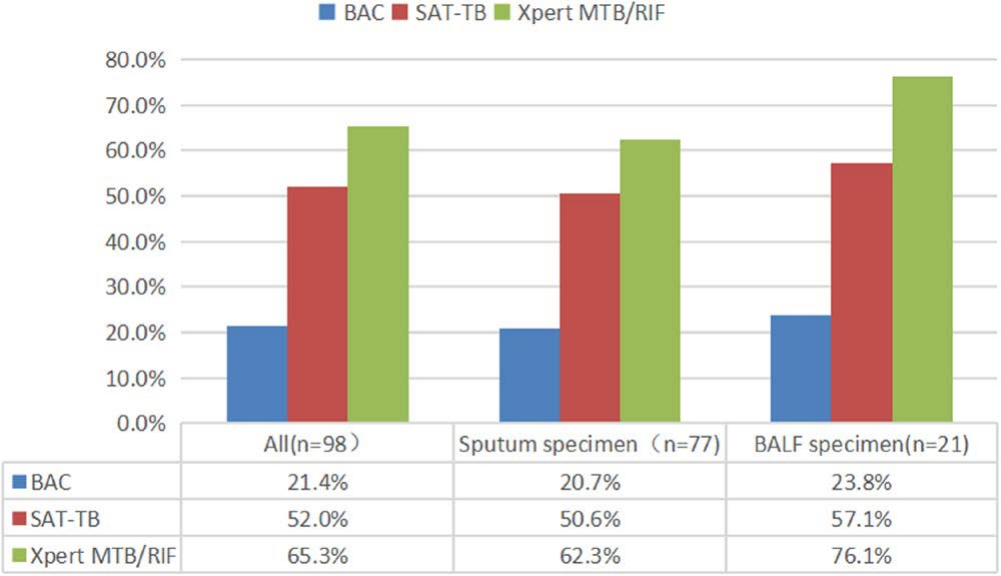
Comparison of the detection of smear-negative PTB by culture, SAT-TB, and Xpert MTB/RIF. MTB/RIF = *Mycobacterium tuberculosis*/rifampin; PTB = pulmonary tuberculosis; SAT-TB = simultaneous RNA amplification and testing method for tuberculosis.

In this study, 114 patients provided 1 sputum specimen each, and 47 patients provided 1 BALF specimen each. The detection rate of SAT-TB in sputum and BALF was significantly higher than that of culture and lower than that of Xpert MTB/RIF. In addition, we observed that the detection rates of the 3 techniques were slightly higher in BALF than in sputum, suggesting that active submission for BALF examination is recommended for patients with conditional BALF examinations (Fig. 2).

### 3.4. Comparison of the diagnostic performance of SAT-TB with that of the other methods and referred by final PTB diagnosis

**Figure 3.**
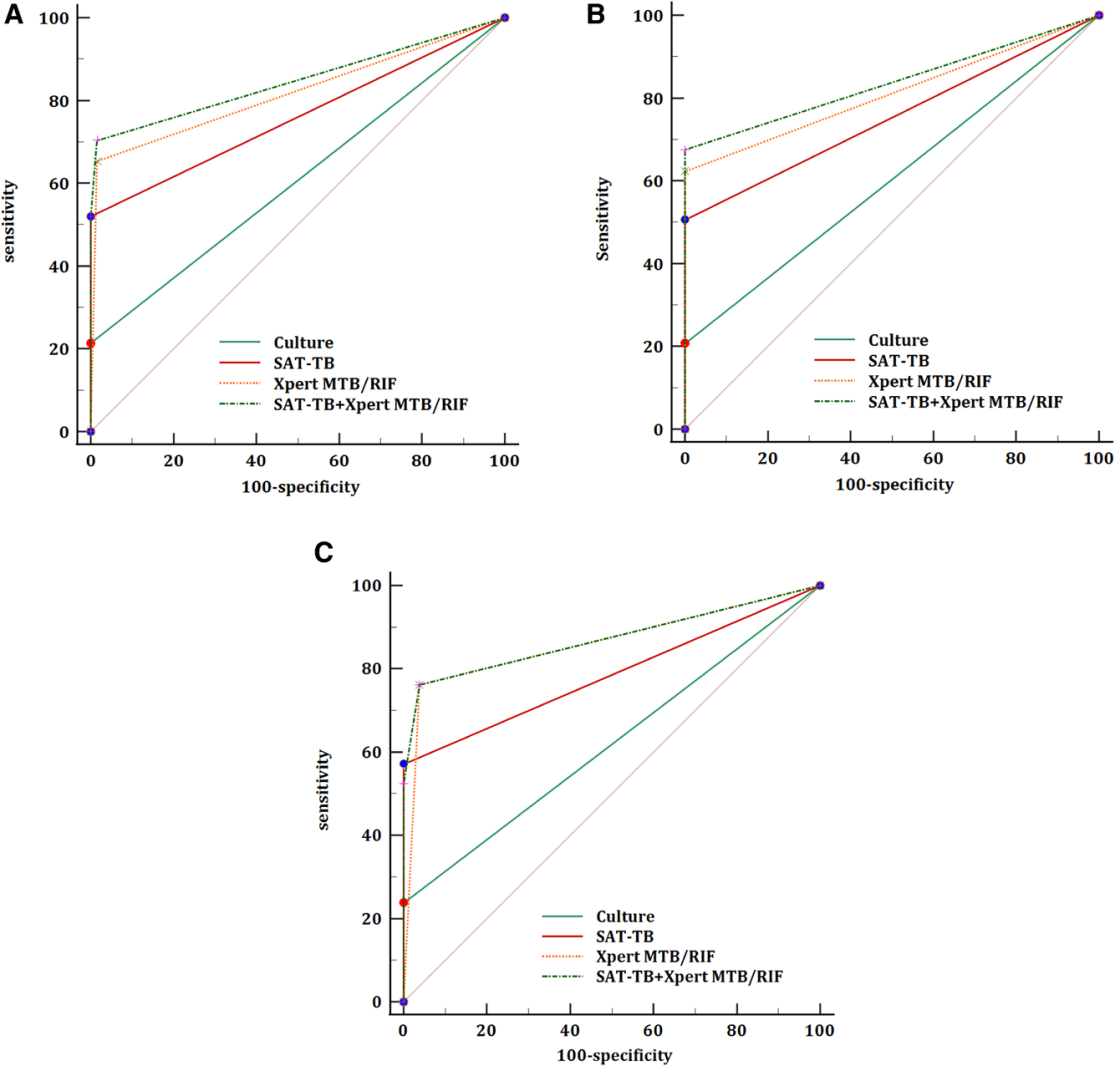
The receiver operating characteristic curves for culture, SAT-TB, Xpert MTB/RIF, and SAT-TB + Xpert MTB/RIF in various respiratory specimens. (A) Sputum and bronchoalveolar lavage fluid; (B) sputum; (C) bronchoalveolar lavage fluid. MTB/RIF = *Mycobacterium tuberculosis*/rifampin; SAT-TB = simultaneous RNA amplification and testing method for tuberculosis.

Cell culture is the gold standard method for PTB diagnosis. However, it has a low positivity rate and sensitivity for smear-negative PTB, making it difficult to meet clinical needs. In this study, when compared with the final diagnosis, the AUC and sensitivity of SAT-TB for smear-negative PTB were 0.76 and 52.0%, respectively, which were higher than those of culture, with an AUC of 0.61 and sensitivity of 21.4% but lower than those of Xpert MTB/RIF, with an AUC of 0.82 and sensitivity of 65.3%. The diagnostic performance for smear-negative PTB can be improved by combining SAT-TB with the Xpert MTB/RIF detection assay (Table 2, Fig. 3A).

**Table 2:** Comparison of the diagnostic efficacy of the 3 tests in smear-negative PTB.

	SAT-TB	Xpert MTB/RIF	Culture	SAT-TB + Xpert MTB/RIF
AUC (95% CI)	0.76 (0.69–0.82)	0.82 (0.75–0.88)	0.61 (0.53–0.68)	0.85 (0.78–0.90)
Sensitivity (%)	52.0 (41.7–62.2)	65.3 (55.0–74.6)	21.4 (13.8–30.9)	70.4 (60.3–79.2)
Specificity (%)	100.0 (94.3–100.0)	98.4 (91.5–100.0)	100.0 (94.3–100.0)	98.4 (91.5–100.0)
PPV (%)	100.0 (93.0–100.0)	98.5 (90.1–99.8)	100.0 (83.9–100.0)	98.6 (90.8–99.8)
NPV (%)	57.3 (52.2–62.2)	64.6 (58.1–70.6)	45.0 (42.5–47.6)	68.1 (61.1–74.4)
Statistical value	1.347			
*P* value	.178			

AUC = the area under the curve, NPV = negative predictive value, PPV = positive predictive value.

### 3.5. Diagnosis efficacy of PTB in sputum and BALF specimens

BALF specimens are favored by clinics because of their strict sampling, high quality, and high reliability of the results. In our study, the AUC and sensitivities of the 3 test techniques (culture, SAT-TB, and Xpert MTB/RIF) for the diagnosis of smear-negative PTB were higher in BALF specimens than in sputum specimens (Table 3, Fig. 3B and C).

**Table 3:** Comparison of diagnostic efficacy of 3 assays in sputum and BALF specimens.

	Sputum specimen (n = 114)				BALF specimen (n = 47)			
	SAT-TB	Xpert MTB/RIF	Culture	SAT-TB + Xpert MTB/RIF	SAT-TB	Xpert MTB/RIF	Culture	SAT-TB + Xpert MTB/RIF
AUC (95% CI)	0.75 (0.66–0.83)	0.81 (0.73–0.88)	0.60 (0.51–0.69)	0.84 (0.76–0.90)	0.79 (0.64–0.89)	0.86 (0.73–0.95)	0.62 (0.47–0.76)	0.87 (0.74–0.95)
Sensitivity (%)	50.7 (39.0–62.2)	62.3 (50.6–73.1)	20.8 (12.4–31.5)	67.5 (55.9–77.8)	57.1 (34.0–78.2)	76.2 (52.8–91.8)	23.8 (8.2–47.2)	76.2 (52.8–91.8)
Specificity (%)	100.0 (90.5–100.0)	100.0 (90.5–100.0)	100.0 (90.5–100.0)	100.0 (90.5–100.0)	100.0 (86.8–100.0)	96.2 (80.4–99.9)	100.0 (86.8–100.0)	96.2 (80.4–99.9)
PPV (%)	100.0 (91.0–100.0)	100.0 (92.6–100.0)	100.0 (79.4–100.0)	100.0 (93.2–100.0)	100.0 (73.5–100.0)	94.1 (69.7–99.1)	100.0 (47.8–100.0)	94.4 (71.1–99.2)
NPV (%)	49.3 (43.7–55.0)	56.1 (48.9–63.0)	37.8 (35.1–40.5)	59.7 (51.8–67.1)	74.3 (63.8–82.6)	83.9 (70.7–91.8)	61.9 (56.1–67.4)	86.2 (72.1–93.8)
Statistical value	1.153				0.880			
*P* value	.249				.379			

AUC = the area under the curve, BALF = bronchoalveolar lavage fluid, NPV = negative predictive value, PPV = positive predictive value.

### 3.6. Comparison of the consistency of SAT-TB and Xpert MTB/RIF results

For sputum specimens, the kappa value of SAT-TB and Xpert MTB/RIF test results was 0.686, with 95% CI (0.551–0.822); for BALF specimens, the kappa value of SAT-TB and Xpert MTB/RIF test results was 0.656, with 95% CI (0.428–0.883); and for all patients in this study, the kappa value of SAT-TB and Xpert MTB/RIF test results was 0.679 with 95% CI (0.563–0.795). For patients with smear-negative suspected PTB, there was a high concordance between the SAT-TB and Xpert MTB/RIF test results for both sputum and BALF specimens.

### 3.7. Patient follow-up results

In this study, 98 patients with a final clinical diagnosis of active PTB were treated with antituberculosis therapy. One patient died after 1 month of follow-up, and the remaining patients showed satisfactory results with antituberculosis treatment.

## 4. Discussion

Smear-negative PTB has a low bacterial load in respiratory tract specimens, making diagnosis difficult, and the missed diagnosis rate is >50%.^[13]^ Therefore, rapid and accurate diagnosis of PTB can provide timely antituberculosis treatment, delay disease progression, reduce respiratory transmission, and improve the prognosis of patients with PTB.^[14–16]^ Although the continuous progress at the technical level has improved the early detection rate of PTB, it is a high-burden disease; moreover, striking a balance between detection efficiency and economic benefits remains a key clinical consideration.

Xpert MTB/RIF is a technique for the rapid diagnosis of PTB by detecting DNA products and has the advantages of high efficiency and sensitivity.^[17,18]^ In our study, the AUC and sensitivity of the Xpert MTB/RIF assay for the diagnosis of smear-negative PTB were significantly higher than those of culture, but slightly higher than that of SAT-TB. In the case of a patient who had previously been diagnosed with PTB and underwent regular antituberculosis treatment, the Xpert MTB/RIF assay tested positive, whereas culture and SAT-TB assay tested negative; the patient’s condition improved after antibacterial treatment, and the final clinical diagnosis was confirmed as inactive PTB. The reason for this discrepancy between different test results is that Xpert MTB/RIF tests for DNA and cannot differentiate between dead and alive bacteria; therefore, by including dead bacteria, the Xpert MTB/RIF test result may become positive. Hence, SAT-TB has more advantages than Xpert MTB/RIF in judging the therapeutic effect of PTB. In addition, 85% of PTB occurs in economically backward areas, whereas Xpert MTB/RIF requires highly technical laboratory equipment and monitoring personnel, is expensive, and increases the economic burden on patients.^[19–21]^

SAT-TB detects RNA products in the diagnosis of PTB, and its diagnostic efficacy is second only to Xpert MTB/RIF.^[22,23]^ Consistent results were obtained in the present study. In addition, SAT-TB involves a less expensive detection technology, a lower price that is less than half that of Xpert MTB/RIF, and can distinguish live bacteria.^[24,25]^ Therefore, SAT-TB is more suitable for the rapid diagnosis of smear-negative PTB in areas with a high tuberculosis burden. In our study, the combined detection using Xpert MTB/RIF and SAT-TB further improved the diagnostic efficiency of smear-negative PTB, and it is a good choice in economically developed areas. In the course of treatment and follow-up of PTB, it is necessary to regularly monitor the etiology, and infectivity, and evaluate the therapeutic effect; however, smear sensitivity is low, culture time is long, Xpert MTB/RIF is expensive, and cannot distinguish live bacteria. Its detection value is limited in the treatment and follow-up of PTB. Therefore, SAT-TB can be used as one of the methods for the treatment and follow-up of PTB.

In addition, previous studies have suggested that BALF may have better diagnostic accuracy and more reliable results for PTB than sputum because it may contain a higher amount and quality of bacterial content and be less susceptible to contamination.^[26,27]^ In this study, the diagnostic efficacy of BALF in smear-negative PTB was slightly higher than that of sputum. This may be due to small sample sizes. Thus, for patients without sputum or an inability to submit qualified sputum specimens, fiberoptic bronchoscopy examination of BALF remains an important means of clinical diagnosis of smear-negative PTB.^[28,29]^

As with all tests, there are some limitations in using SAT-TB for detecting smear-negative suspected PTB cases. The first is RNA degradation. RNA products are more unstable than DNA amplification products outside the reaction tube. In practical operations, RNA is easily degraded by various factors during sample collection, preservation, and transportation, which affects the sensitivity and accuracy of SAT-TB detection.^[12]^ Second, the relatively small number of live MTBs in smear-negative patients with suspected PTB results in the production of a small amount of RNA, which may affect the test results. Thirdly, during the SAT-TB detection process, the operator’s skills and experience may affect the results. In addition, while a multicenter prospective study can help reduce bias, it has certain limitations, such as a small sample size and the inability to achieve standardization in sample collection, sampling, and testing procedures. Given these limitations, it is imperative to establish rigorous standards for sample collection, storage, and processing to minimize the risk of RNA degradation. Implementing quality control measures is also essential to ensure consistency and accuracy throughout the detection process.

In summary, SAT-TB has the advantages of high sensitivity and specificity, simple operation, absence of temperature cycling, high amplification efficiency, short reaction time, low price, and high concordance with Xpert MTB/RIF for the diagnosis of smear-negative suspected PTB and still shows high detection efficacy in both sputum specimens and BALF. Therefore, SAT-TB is of high clinical value for early and rapid diagnosis of smear-negative suspected PTB in resource-limited areas. At the same time, it has more advantages in evaluating the effect of treatment and judging the recurrence of PTB. Additionally, to enhance the role of SAT-TB technology in global tuberculosis control efforts, future research can be conducted in the following areas: Firstly, to refine the SAT-TB technique, improving its stability and reproducibility while reducing the incidence of false positives and false negatives; secondly, to develop an automated SAT-TB detection platform to minimize human error and enhance detection efficiency; thirdly, to conduct multicenter clinical trials of SAT-TB in various regions and populations to substantiate its broad applicability and efficacy; fourthly, to investigate the diagnostic performance of SAT-TB in different patient groups (such as HIV-infected individuals, children, the elderly, and patients with drug-resistant tuberculosis, etc), and to explore whether adjustments to detection parameters are needed for specific groups; and lastly, to integrate SAT-TB technology into current guidelines for tuberculosis screening and diagnosis, complementing existing diagnostic methods to form a more comprehensive and efficient strategy for tuberculosis diagnosis.

## 5. Conclusion

For different respiratory tract specimens, the diagnostic efficacy of SAT-TB in smear-negative suspected PTB was moderate, which was significantly better than that of culture but inferior to that of Xpert MTB/RIF, and was highly consistent with Xpert MTB/RIF in terms of diagnostic accuracy. It is a valuable method for early detection, prevention, and managing smear-negative PTB suspects. Meanwhile, the detection efficiency and cost-effectiveness of SAT-TB are more suitable for the rapid diagnosis of smear-negative PTB in low- and middle-income countries.

## Acknowledgments

We wish to thank all the patients who participated in this study.

## Author contributions

**Conceptualization:** Xiaoxiao Zhao, Lang Bai, Wei Liu, Rili MiSe.

**Formal analysis:** Lingcheng Cheng.

**Funding acquisition:** Lang Bai, Shanling Xu.

**Investigation:** Xiaoxiao Zhao, Kunping Cui, Wei Liu.

**Methodology:** Kunping Cui.

**Project administration:** Wen Quan Li, Chuan Zhao.

**Resources:** Lang Bai, Jin Shang.

**Supervision:** Shanling Xu.

**Software:** Jin Shang.

**Validation:** Shanling Xu, Lin Wang.

**Visualization:** Wen Qiu Deng.

**Writing – original draft:** Xiaoxiao Zhao.

**Writing – review & editing:** Xiaoxiao Zhao, Kunping Cui, Lang Bai.
